# Postprandial muscle protein synthesis is higher after a high whey protein, leucine-enriched supplement than after a dairy-like product in healthy older people: a randomized controlled trial

**DOI:** 10.1186/1475-2891-13-9

**Published:** 2014-01-22

**Authors:** Yvette C Luiking, Nicolaas EP Deutz, Robert G Memelink, Sjors Verlaan, Robert R Wolfe

**Affiliations:** 1 Nutricia Advanced Medical Nutrition, Nutricia Research, Utrecht, the Netherlands; 2 Center for Translational Research in Aging and Longevity, Department of Health & Kinesiology, Texas A&M University, College Station, TX, USA; 3 Center for Translational Research in Aging and Longevity, Donald W. Reynolds Institute on Aging, University of Arkansas for Medical Sciences, 4301 W. Markham St. Slot 806, Little Rock AR 72205, USA

**Keywords:** Muscle, Protein synthesis, Older people, Leucine, Whey, Exercise

## Abstract

**Background:**

Decreased ability of muscles to respond to anabolic stimuli is part of the underlying mechanism for muscle loss with aging. Previous studies suggest that substantial amounts of essential amino acids (EAA), whey protein and leucine are beneficial for stimulation of acute muscle protein synthesis in older adults. However, these studies supplied only proteins, and no bolus studies have been done with dairy products or supplements that contained also fat and carbohydrates besides proteins. The aim of this study was to evaluate whether a specifically designed nutritional supplement in older adults stimulates muscle protein synthesis acutely to a greater extent than a conventional dairy product. Moreover, the combined effect with resistance exercise was studied by using a unilateral resistance exercise protocol.

**Methods:**

Utilizing a randomized, controlled, double blind study design, healthy older adults received a single bolus of a high whey protein, leucine-enriched supplement (EXP: 20g whey protein, 3g total leucine, 150kcal; n = 9) or an iso-caloric milk protein control (Control: 6g milk protein; n = 10), immediately after unilateral resistance exercise. Postprandial mixed muscle protein fractional synthesis rate (FSR) was measured over 4h using a tracer infusion protocol with L-[ring-^13^C_6_]-phenylalanine and regular blood and muscle sampling.

**Results:**

FSR was significantly higher overall after EXP (0.0780 ± 0.0070%/h) vs Control (0.0574 ± 0.0066%/h (EMM ± SE)) (p = 0.049). No interaction between treatment and exercise was observed (p = 0.519). Higher postprandial concentrations of EAA and leucine are possible mediating factors for the FSR response, while plasma insulin increase did not dictate the FSR response. Moreover, when the protein intake from the supplements was expressed per kg leg lean mass (LLM), a significant correlation was observed with resting postprandial FSR (r = 0.48, *P* = 0.038).

**Conclusions:**

Ingestion of a high whey protein, leucine-enriched supplement resulted in a larger overall postprandial muscle protein synthesis rate in healthy older subjects compared with a conventional dairy product. This acute effect is promising for long-term effects on parameters of muscle mass, strength and function in sarcopenic older people, which requires further study.

**Trial registration:**

This trial is registered in the Dutch Trial Register under number NTR1823.

## Background

Muscle mass, strength and function decreases with aging, and can evolve into sarcopenia, i.e. low muscle mass and function, if the loss is sufficient [1]. It has been hypothesized that a decreased ability of the muscle to respond to anabolic stimuli, such as essential amino acids (EAA) and leucine in a meal [2-4] or exercise [5,6], is part of the underlying mechanism explaining the muscle decrements in older adults.

Stable isotope studies have demonstrated that ingestion of amino acids can stimulate muscle protein synthesis in older adults [7-9]. EAA in particular are able to stimulate muscle protein synthesis, whereas non-EAA (NEAA) are of less relevance for muscle anabolism [9,10]. However, older adults required a higher dose of EAA to stimulate muscle anabolism than younger individuals [7,11]. This suggests that the anabolic response to amino acids at low doses is blunted in older people [3,12]. In addition to increasing the total dose of amino acids, diminished anabolic responsiveness with aging could also be overcome by increasing the proportion of leucine [12], especially at low protein intake [13]. Moreover, a protein source such as whey, that is digested rapidly and absorbed with a rapid elevation of plasma leucine, could stimulate muscle protein synthesis rate to a higher level than casein or casein hydrolysate [14,15]. Therefore, dietary strategies with a mixture of whey protein and additional leucine that significantly increase plasma EAA and leucine are hypothesized to be beneficial for a greater acute muscle anabolic response in older adults than other proteins alone and in an amount that is present in a conventional dairy product.

Resistance exercise is another stimulus for muscle protein synthesis. However, since exercise also activates muscle protein breakdown, net muscle protein balance remains negative, i.e. with net protein breakdown, in the absence of nutrient intake [16]. The combination of exercise prior to amino acid supply enhanced muscle protein synthesis further than exercise alone and resulted in net muscle protein synthesis [17,18]. The interactive effect of amino acids and exercise was likely due in part to exercise-stimulated blood flow and increased amino acid delivery to the muscle [18]. It is also possible that activation of the mTOR (mammalian target of rapamycin) pathway by exercise primed the muscle to respond more prominently than when amino acids were ingested alone [19]. On the other hand, older adults apparently have impaired contraction-induced activation of mTOR signaling and muscle protein synthesis [5,6]. Nonetheless, protein or amino acids in combination with exercise has additive effects on muscle protein synthesis in older adults although the response may be somewhat delayed with aging [20].

The primary objective of our study was to evaluate muscle protein synthesis in healthy older adults after intake of a specifically designed high whey protein, leucine-enriched nutritional supplement, compared with a control product containing milk protein in an amount that is typical for a single serving of a conventional dairy product, e.g., a glass of milk. Both products contained besides protein also carbohydrates and fat and thus are more representative of conventional products or a ‘mixed meal’, in contrast to previous studies in which only intact protein or free amino acids were supplied. To account for a potential impact of energy density, both products were isocaloric. Secondly, the combined effect on muscle protein synthesis of nutritional supplementation with resistance exercise was compared to nutritional supplementation without exercise, by using an unilateral resistance exercise protocol. We hypothesized that the nutritional supplement high in whey protein and enriched in leucine would be more effective than a typical dairy product to stimulate muscle protein synthesis in healthy older adults. Moreover, we hypothesized that exercise would further enhance postprandial muscle protein synthesis. These objectives are relevant for the ultimate use of nutritional supplementation and exercise intervention in the management and prevention of sarcopenia.

## Methods

### Subjects

**Figure 1:**
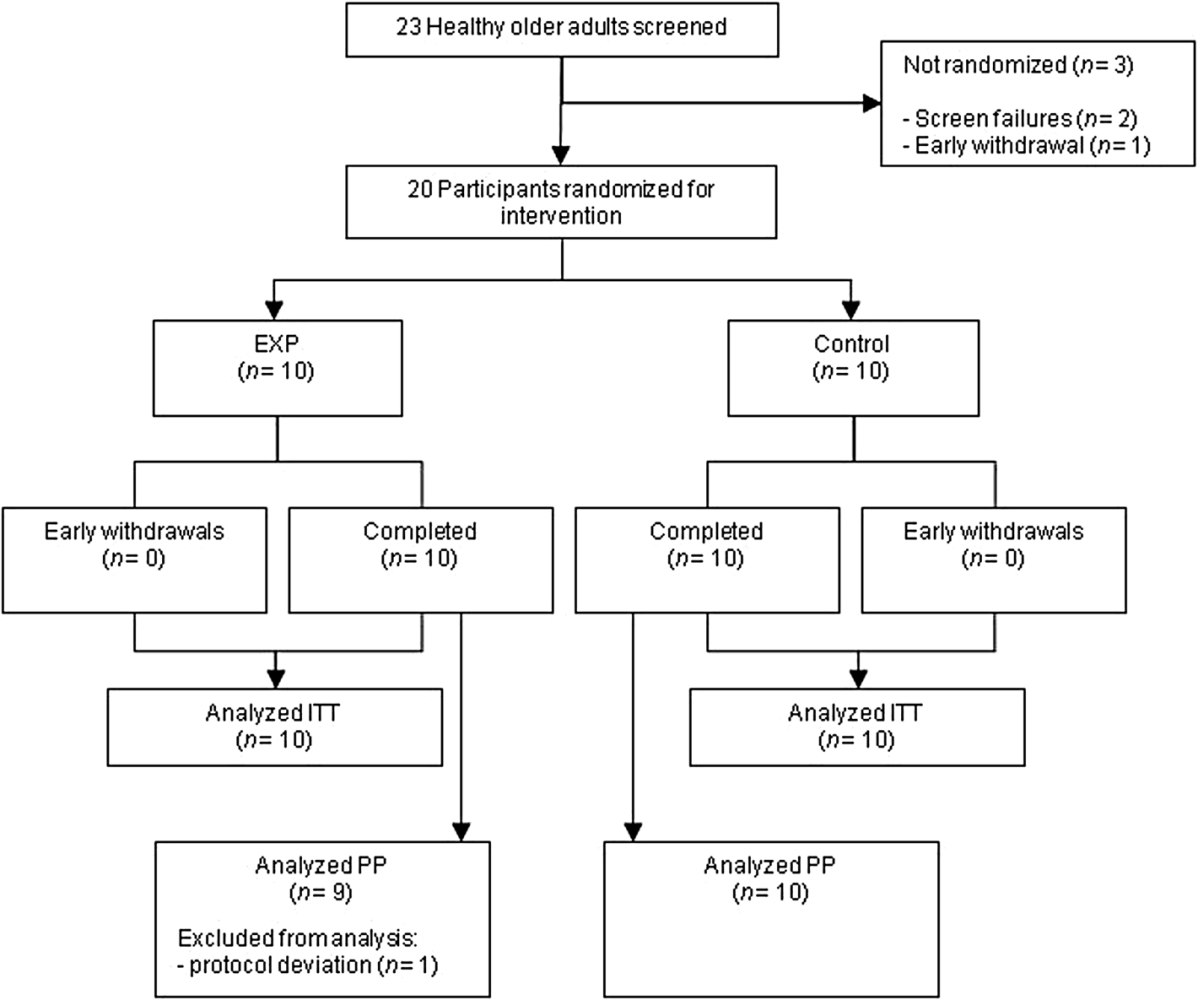
**Flow diagram.** Flow diagram of participants.

Twenty-three healthy older adults were screened and a total of 20 healthy older adults were enrolled in the study. All participants were 60 y or older with a Body Mass Index (BMI) between 21–30 kg.m^-2^, were able to walk, sit down and stand up independently and had the ability to sign informed consent. Men and women were excluded from participation if they had any (history of) gastrointestinal disease, were diagnosed and actively treated for diabetes mellitus type I or II, had a history of congestive heart failure or recent hospitalization for heart disease or myocardial infarction, or had a history of hypoglycaemia. Other exclusion criteria were: systolic blood pressure >180 mmHg and/or diastolic blood pressure >110 mmHg, infection or fever in the last 7 d, medication use (antibiotics within 3 wk prior to study visit, current use of corticosteroids, growth hormone or testosterone), known allergy to milk and milk products, lactose intolerance and known galactosaemia, as well as blood haemoglobin <9 g.dL^-1^. Moreover, those with platelet count <100,000.mL^-1^, a history of hypo- or hyper-coagulation disorders including use of a coumarin derivative, history of deep venous thrombosis, or pulmonary embolism at any point in lifetime were also excluded. No participants were involved in a weight loss or muscle strengthening program, used protein containing or amino acid containing nutritional supplements (within one week of study entry), smoked (for the past 3 mo) or abused alcohol or drugs at time of screening and study visit. The Modified Baecke Questionnaire for Older Adults [21] was filled out to record the habitual physical activity level. Figure 1 shows the flow diagram for participants.

All participants were informed of the procedures involved and all possible risks before their informed consent was obtained. The study was conducted according to ICH-GCP principles, and in compliance with the principles of the ‘Declaration of Helsinki’ (59th WMA General Assembly, Seoul, October 2008) and the local laws and regulations of the country where the study was performed. The study was approved by the Institutional Review Board at the University of Arkansas for Medical Sciences.

### Design

**Figure 2:**
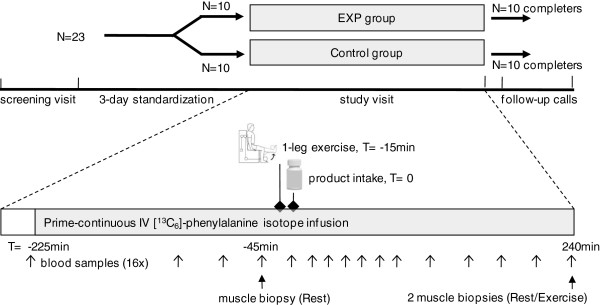
**Study design.** Study design, including a screening visit, a 3-d standardization period, study visit and follow-up calls. At the study visit, participants underwent a stable isotope infusion protocol for 7¾ h, including an initial fasting period of 3¾ h until product intake (T = 0), regular blood sampling (arrows), 3 muscle biopsies (T = -45 and 240 min), and an unilateral resistance exercise intervention (T = -15 min).

A randomised, controlled, double-blind, parallel groups, single-centre study design was applied. After having signed for informed consent at screening visit, participants underwent a blood draw for Complete Blood Count analysis. Eligible participants underwent a DEXA-scan (dual energy x-ray absorptiometry), and determination of 1-RM (1-repetition maximum) for leg extension, and received instructions and a diet diary for the 3-d standardization phase preceding the study visit. At the study visit, participants were randomly allocated to receive one of the two study products, using stratification for sex to account for possible sexual dimorphism in muscle protein synthesis response [22]. A 7¾-h isotope infusion protocol with sampling of muscle biopsies and blood was followed to determine postprandial mixed muscle protein synthesis over 4 h after bolus intake of the study product. A unilateral leg resistance exercise protocol, performed 15 min before ingestion of the nutritional product, enabled measurement of muscle protein synthesis in both the resting and the exercising leg. Two follow-up phone calls, at 1–3 d and 5–10 d after the study visit, were scheduled for identification of adverse events and evaluation of tolerance. Use of relevant concomitant medication and nutritional supplements was recorded throughout the study. Figure 2 shows a schematic representation of the study design. Study staff and participants were blinded to the study products until completion of the study database including all laboratory data.

### Standardization phase

Participants were advised to adhere to their regular dietary intake, except from fasting state from 22:00 h the day prior to the study visit. They were also asked not to take part in any feast with excessive amounts of food for the 3 d prior to the study visit. Participants were requested not to use alcohol within 24 h prior to the study visit, and not to perform intense physical activities (i.e. sports, heavy gardening and housekeeping, and walking or cycling longer than normal) for 72 h prior to the study visit. Dietary intake was calculated based on the 3-d diet diary and reviewed by a dietician. The mean daily dietary protein intake was estimated using a computer programme (The Food Processor, ESHA Research). Portion size reported by the participants was evaluated based on pictures participants made of their meals.

### Anthropometric measurements

Body height, body weight and body composition were measured at the day of enrollment. Body weight and height were used to calculate BMI. Body composition, i.e. whole body fat mass, lean body mass and lean mass of both legs (leg lean mass, LLM), was determined by DEXA-scan using a Delphi DEXA scanner (QDR 4500W, Hologic Inc., Bedford, MA).

### Study products

Both study products, i.e. Experimental and Control, provided 150 kcal (36 kJ) in a dose of 200 mL (Table 1). The experimental study product (EXP) consisted of a high whey protein, leucine-enriched nutritional supplement (20 g whey protein, 3 g total leucine). The control study product (Control) contained an amount of milk protein (6 g protein) that is common for one serving of a conventional dairy product, e.g. a glass of milk. The amount and source of fat in the products were similar. The amount of carbohydrates in the Control product was higher and added as maltodextrin (19 DE). The osmolality of the EXP product was slightly higher (400 mOsm.kg^-1^) compared with the Control product (350 mOsm.kg^-1^). The product had to be consumed completely as a single bolus, and all participants completed consumption within 5 min. Study products were labelled with randomization numbers, and independent site staff mixed the study products (as powders) with water to a volume of 200 mL prior to consumption.

**Table 1:** Composition of the study products

**Component**		**Unit**	**EXP product**	**Control product**
Energy		Kcal	150	150
	Protein	%	56	16
	Carbohydrates	%	26	66
	Fat	%	18	18
Protein	Total	g	21	6^1^
Intact protein	Whey	g	20	1.2
	Casein	g	0	4.8
Free amino acid	BCAA	g	1	0
Total leucine^2^		g	3	0.6
Total EAA^2^		g	10.6	3.0
Total phenylalanine		g	0.6	0.3
Carbohydrates	Total	g	10	25
	Sucrose	g	7.5	7.4
	Glucose	g	0	0.3
	Maltose	g	0	1.1
	Maltodextrin	g	2.2	15.9
	Lactose	g	0.05	0.07
Fat	Total	g	3	3
	Palm oil	g	2.9	2.9
	Milk fat	g	0	0.1

BCAA, branched chain amino acids; EAA, essential amino acids.

The study products contain no vitamins, trace elements, minerals, or other additions.

^1^ Provided as milk protein.

^2^ Provided by protein and free BCAA.

### Isotope infusion protocol

Participants reported to the lab in the morning of the study visit after an overnight fast. An 18–22 gauge catheter was placed into veins of the right and left forearms for blood sampling and stable isotope infusion. All timings relate to the time of product intake as T0. Baseline blood samples were collected for fasting concentrations of serum C-reactive protein (CRP), albumin, and pre-albumin, and background plasma amino acid enrichment (T = -225 min). Thereafter, the plasma phenylalanine pool was primed with a single intravenous priming dose (4.2 μmol.kg^-1^) of L-[ring-^13^C_6_]-phenylalanine (Cambridge Isotope Labs, Andover, MA). This was followed immediately by a continuous infusion (0.07 μmol.kg^-1^.min^-1^) of L-[ring-^13^C_6_]-phenylalanine and maintained throughout the experiment. Blood was taken from the sampling forearm at T = -105, -75, and -45 min, respectively, for the determination of plasma phenylalanine enrichment and concentrations of amino acids, glucose and insulin. A first muscle biopsy was taken at T = -45 min from the non-dominant leg. Unilateral resistance exercise with the dominant leg was performed at T = -15 min with the non-dominant leg in rest. Immediately following the exercise protocol, a blood sample was taken followed by bolus ingestion of the study product (200 mL at T0). Blood samples were then drawn over the next 4 h, at 15, 30, 45, 60, 75, 90, 120, 150, 180, 210, 240 min after start of product intake for analyses of amino acid enrichments, and concentrations of amino acids, glucose and insulin. Two more muscle biopsies, one from each leg, were taken at 4 h after start of product intake at T = 240 min.

The participants lay in bed throughout the study except when they had to use the bathroom and during the exercise protocol. Vital signs (blood pressure, heart rate, body temperature and respiration rate) were recorded once during the study visit, at baseline. Muscle biopsies (50–100 mg) were taken under local anesthetic from the lateral portion of the vastus lateralis, approximately 10–15 cm above the knee, using a 5 mm Bergstrom biopsy needle (Stille, Stockholm, Sweden). Muscle samples were used to measure protein-bound phenylalanine enrichment (as molar percent excess, MPE) for the purpose of calculating mixed muscle protein fractional synthetic rate (FSR). A tolerance questionnaire was taken before product ingestion and at the end of the experiment to record intensity of gastrointestinal symptoms (heartburn, belching, nausea, vomiting, abdominal distension, flatulence, diarrhea, and constipation), light-headedness, headache, thirst, and dry mouth.

### Unilateral leg resistance exercise protocol

Leg extensions were performed in seated position thru a full range of motion using Keiser pneumatically controlled machines with a starting angle of 110° of flexion. The lower part of the dominant leg was conveniently placed against the bar of the extender arm of the machine. Participants performed a short warming-up of 5–10 unloaded leg extensions, followed by 10 extensions at 40% of subject’s 1-RM, as determined during the screening visit. During a short break of maximum 2 min, the load was set at 80% of the subject’s 1-RM. Participants performed 4 sets of 8–10 repetitions of leg extensions at 80% of 1-RM, with counter-resisted flexion movement back to the starting position. Duration ratio of extension and flexion movement was 1:2. Resting interval between sets was 2 min. The protocol lasted about 15 min. A trained instructor supervised the exercise procedure.

### Sample processing and analyses

Blood samples were collected in pre-chilled heparinized tubes (Becton Dickinson Vacutainer system, Franklin Lakes, New Jersey, USA) and kept on ice to minimize enzymatic reactions. All plasma samples were stored at -80°C until further analyses. Muscle samples were prepared and processed as previously described [23].

Muscle free intracellular, protein-bound, and plasma L-[ring-^13^C_6_]-phenylalanine enrichments were determined using the tert-butyldimethylsilyl derivative and GCMS (Agilent Technologies model 5973) with electron impact ionization as described previously [23]. Glucose concentrations were measured using an YSI glucose auto-analyzer. Amino acid concentrations in plasma were measured by liquid chromatography-mass spectrometry using the internal standard approach for each individual amino acid, as previously described [23]. Serum CRP and insulin concentrations were measured by ELISA. Serum albumin and pre-albumin were analyzed using validated methods.

### Calculations

The tracer-tracee ratio (t/T) was calculated from the difference in the area counts of the mass fragments from 234 and 240. Enrichment as MPE was then calculated as (t/T)/(t/T + 1).

The primary endpoint of the study was the FSR (in %.h^-1^) of mixed muscle protein. FSR is calculated by dividing the increment in the enrichment in muscle protein-bound ^13^C_6_-phenylalanine over time (∆MPEp) by the enrichment of the precursor and the length of time between biopsies. The mean plasma enrichment (MPE as area/time) of ^13^C_6_-phenylalanine (Eplasma) between the two biopsies was considered as the most representative precursor under this non-steady state situation, anticipating that the different phenylalanine content of the study products would affect steady-state differently. Even more so because no L-[ring-^13^C_6_]-phenylalanine was added to the study products to keep the blinded character of the study. This approach has also been used in previous studies with bolus product intake, yielding comparable results to the traditional intracellular precursor enrichment for calculation of FSR, although absolute FSR levels are generally lower with the plasma precursor [14,23]. FSR was thus calculated as (∆MPEp)/(Eplasma*t) * 60 *100, where t represents the time interval between the two biopsies and the factors 60 and 100 are used to express FSR values in percent per hour. FSR was calculated separately for the resting leg and the exercising leg, based on the increment in enrichment from the first biopsy to the second biopsy in either of the 2 legs.

Since only one biopsy was taken in the postabsorptive state, basal FSR could not be calculated according to the traditional 2-biopsy method. In an attempt to calculate basal FSR, the so-called single-biopsy calculation was applied [24] by using above equation of FSR where ∆MPEp now represents the difference in ^13^C_6_-phenylalanine enrichment between (a) muscle bound mixed protein from the muscle biopsy taken at 180 min after the start of tracer infusion and (b) plasma albumin protein from the plasma sample taken before the start of tracer infusion and reflecting background or natural protein enrichment. However, since 6 out of 20 participants had participated in a previous tracer infusion study, and recent publications indicate that nontracer naive state results in unreliable muscle protein FSR [24], we decided to not include basal FSR values in the statistical analyses. Therefore, the analysis on the primary parameter FSR was performed on the postprandial data only, unadjusted for basal FSR. Basal FSR values, calculated with data from tracer naive participants, are only used as a reference to estimate the increase in FSR after product intake.

For plasma leucine and EAA (sum of histidine, isoleucine, leucine, lysine, methionine, phenylalanine, threonine, tryptophan, and valine), the maximum (or peak) plasma concentration, the maximum increment from baseline and the iAUC (from baseline, during 4 h after product intake) were calculated. For plasma AA (i.e. sum of all amino acids), the iAUC was calculated. NEAA was calculated as AA minus EAA. The postabsorptive amino acid concentrations were only measured at -45 min, but will be indicated as T0. For plasma glucose and insulin the maximum (or peak) and iAUC values were calculated.

### Statistical analysis

Mixed model analysis was used to compare postprandial FSR values between the two groups; the model included fixed effects for treatment, exercise and sex, a fixed interaction effect for treatment * exercise and subject as random factor. To compare postprandial values for other parameters, the mixed model additionally included the baseline pre-exercise values as a covariate. The check for possible confounding factors was performed for the primary parameter by including one-by-one (independently) BMI, body fat mass, LLM, age, physical activity, mean daily dietary protein intake, or serum CRP concentration in the basic model. For amino acids, glucose and insulin, the serum CRP concentration was added to the basic model as a potential confounder. A check for possible mediating factors was performed for the primary parameter by including one-by-one (independently) iAUC for AA, EAA, NEAA, leucine, glucose, and insulin, as well as maximum concentrations for EAA and leucine in the basic model and comparing the effect size of the treatment effect in the model versus the effect size in the model without possible mediator [25]. Pearson’s correlation coefficients were calculated to analyse a correlation between variables. Sample size calculation (10 per group) was based on an anticipated difference in change in FSR from baseline between EXP (50% estimated increase) and Control group (11% estimated increase), using a variance (SD^2^) of difference of 0.0003%/h (based on [10,12]), an average within subject correlation over time (ρ) of 0.7, an intra-class correlation coefficient (ICC) for nested structure of 0.05, a significance level α of 0.05, and a power of 80%.

SAS 9.1.3 (SAS Institute) software was used for all statistical analyses. Data are expressed as means with SEM, unless otherwise stated. Estimated marginal mean (EMM) with SE, derived from the mixed model, is used for overall analysis. Statistical significance was defined as a two-tailed *P* < 0.05.

## Results

### Subjects

One subject was excluded from the data analysis because of high plasma glucose concentration at baseline, which increased further after product intake; all plasma glucose values for that subject were above 8.3 mmol.L^-1^ (or 150 mg.dL^-1^). This was considered a major protocol deviation. Descriptive and statistical analyses are described for the remaining 19 participants (per-protocol analyses). No significant differences were present in demographics, body composition, screening parameters, and pre-study dietary protein intake between the two groups (Table 2). The participants in the two groups did not differ in their medical history, and no clinically relevant differences in adverse events and gastrointestinal symptoms were observed between both groups.

**Table 2:** **Demographic data and other subject characteristics at baseline (per-protocol population)**^**1**^

**Characteristic**	**EXP (*n*** **= 9)**	**Control (*n*** **= 10)**	**Total (*n*** **= 19)**	***P *** **value**^**2**^
Male/female, *n*	4/5	5/5	9/10	1.00
Age, *y*	66.9 ± 4.8	71.1 ± 6.3	69.1 ± 5.9	0.12
Body weight, *kg*	79.90 ± 12.23	78.75 ± 13.89	79.29 ± 12.78	0.85
BMI, *kg.m*^*-2*^	27.08 ± 1.88	26.14 ± 2.59	26.58 ± 2.27	0.38
Fat mass, *kg*	25.88 ± 4.62	25.72 ± 8.21	25.80 ± 6.57	0.96
Lean body mass, *kg*	49.17 ± 10.07	48.11 ± 10.04	48.61 ± 9.79	0.82
Leg lean mass^3^, *kg*	15.83 ± 3.75	15.30 ± 3.91	15.55 ± 3.74	0.77
Serum albumin, *g.L*^*-1*^	2.782 ± 0.335	3.033 ± 0.464	2.914 ± 0.417	0.20
Serum pre-albumin, *g.L*^*-1*^	0.285 ± 0.132	0.252 ± 0.152	0.268 ± 0.140	0.63
Serum CRP, *mg.L*^*-1*^	1.932 ± 1.532	1.620 ± 1.534	1.768 ± 1.498	0.66
Physical activity level, *total score*	7.526 ± 2.766	11.363 ± 5.688	9.545 ± 4.843	0.08
1-RM for single leg extension, *Nm*	96.3 ± 38.7	76.3 ± 25.7	85.8 ± 33.2	0.20
Dietary protein intake, *g.kg BW*^*-1*^*.day*^*-1*^	0.8689 ± 0.1913	1.0136 ± 0.2358	0.9451 ± 0.2226	0.16
Dietary protein intake, *%*	15.96 ± 2.48	17.97 ± 2.99	17.02 ± 2.88	0.14

^1^ All values are means ± SD, except for sex.

^2^*P* value is based on two-sample *t*-test, except for sex a Fisher exact test is used.

^3^ Sum of both legs.

### Plasma amino acids, glucose and insulin

**Figure 3:**
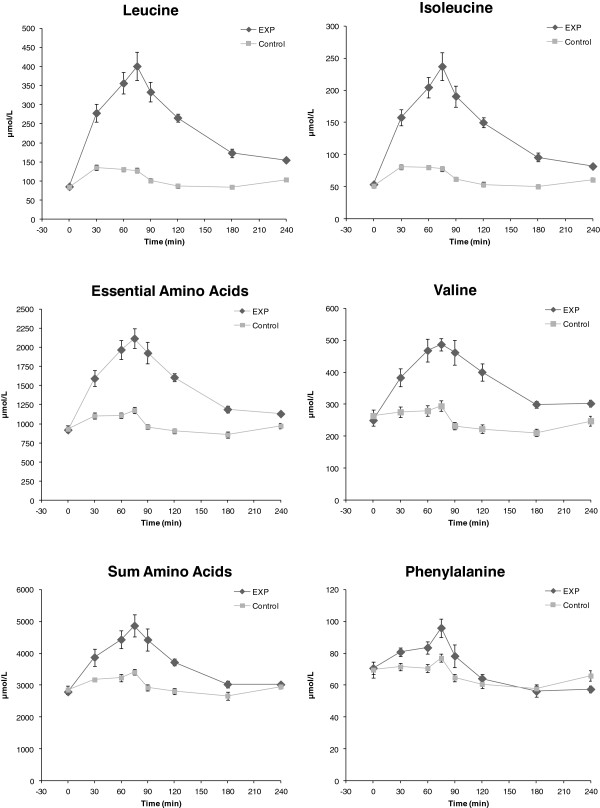
**Plasma amino acids.** Plasma concentrations (means ± SEM) of leucine, essentials amino acids (EAA), sum of amino acids (AA), isoleucine, valine and phenylalanine before and after ingestion (at T = 0) of the study products EXP (*n* = 9) and Control (*n* = 10). Baseline values were not different between groups. iAUC values for leucine, EAA and AA were significantly higher after ingestion of EXP vs Control (*P* < 0.001). For leucine and EAA, peak and change-from-baseline concentrations were also significantly higher after ingestion of EXP vs Control (*P* < 0.001).

Plasma concentrations of leucine, EAA, and AA over time are shown in Figure 3. Baseline values were not different between groups (*P* > 0.70). EXP product intake resulted in a significantly higher peak plasma leucine concentration (406 ± 35 μmol.L^-1^) than intake of the Control product (142 ± 5 μmol.L^-1^; *P* < 0.001). Change in leucine was also significantly higher for EXP (321 ± 34 μmol.L^-1^) vs Control (58 ± 6 μmol.L^-1^) (*P* < 0.001), as was iAUC leucine (40471 ± 2655 and 6344 ± 971 μmol.L^-1^.min for EXP and Control respectively; *P* < 0.001). Peak plasma EAA concentration was significantly higher in EXP (2227 ± 139 μmol.L^-1^) vs Control (1180 ± 33 μmol.L^-1^)(*P* < 0.001), as was change in EAA (1306 ± 148 and 249 ± 32 μmol.L^-1^ for EXP and Control respectively; *P* < 0.001), and iAUC EAA (142281 ± 11931 and 17312 ± 2638 μmol.L^-1^.min^-1^ for EXP and Control respectively; *P* < 0.001). For AA, iAUC was significantly higher after ingestion of EXP (206229 ± 23889 μmol.L^-1^.min^-1^) than Control (39244 ± 11490 μmol.L^-1^.min^-1^)(*P* < 0.001).

**Figure 4:**
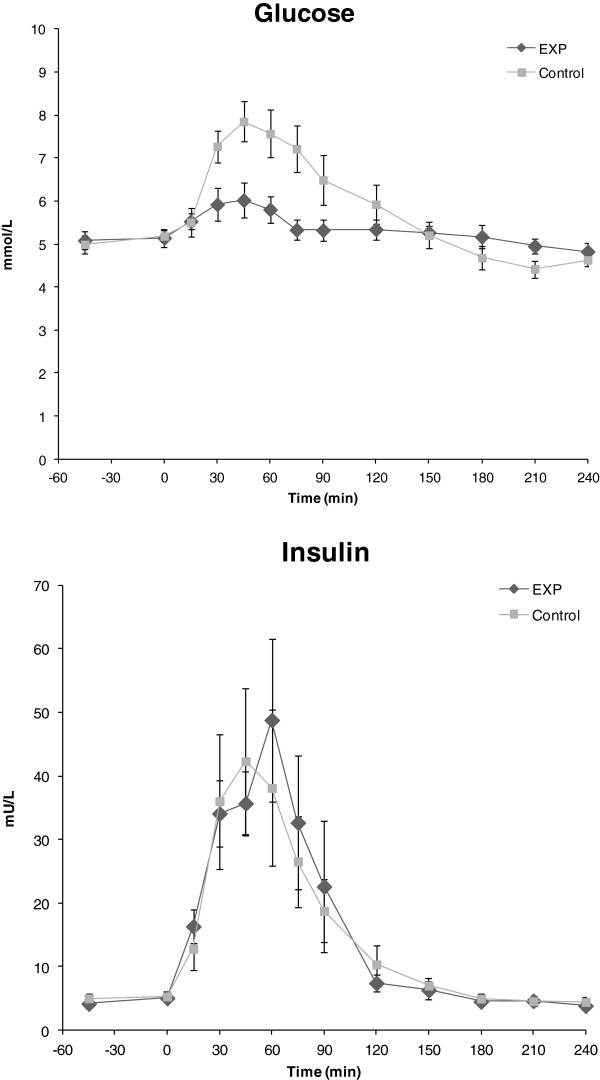
**Plasma glucose and insulin.** Plasma concentrations (means ± SEM) of glucose and insulin before and after ingestion (at T = 0) of the study products EXP (*n* = 9) and Control (*n* = 10). Baseline values were not different between groups. Peak glucose (*P* < 0.001) and glucose iAUC (*P* = 0.003) was significantly higher after ingestion of Control, but groups did not differ significantly in peak or iAUC insulin.

Plasma concentrations of insulin and glucose over time are shown in Figure 4. Baseline glucose was not different between EXP and Control, both before and after exercise (both *P* > 0.60). Ingestion of the Control product increased plasma glucose concentration to a significantly higher peak concentration (8.5 ± 0.4 mmol.L^-1^) than did the EXP product (6.4 ± 0.3 mmol.L^-1^)(*P* < 0.001). iAUC glucose was also higher for Control (246 ± 37 mmol.L^-1^.min^-1^) vs EXP (88 ± 15 mmol.L^-1^.min^-1^) (*P* = 0.003). No significant differences between EXP and Control were observed for baseline insulin either before exercise (*P* = 0.30) or after exercise (*P* = 0.19), and for peak plasma insulin (*P* = 0.17) and iAUC insulin (*P* = 0.17).

### L-[ring]-^13^C_6_-phenylalanine enrichments

**Figure 5:**
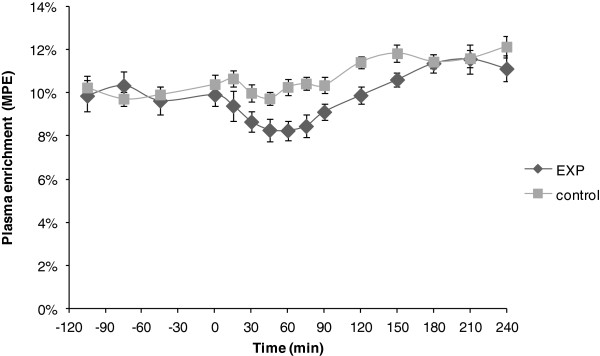
**Plasma **^**13**^**C**_**6**_**-Phe enrichment.** Plasma enrichment of L-[ring-^13^C_6_]-phenylalanine (means ± SEM) expressed as molar percent excess (MPE), before and after ingestion (at T = 0) of the study products EXP (*n* = 9) and Control (*n* = 10).

Plasma enrichment of L-[ring-^13^C_6_]-phenylalanine showed a stable level (steady state) before ingestion of the study products (Figure 5). The profiles suggested that product ingestion caused a disruption in steady state of plasma L-[ring-^13^C_6_]-phenylalanine enrichment.

Mixed muscle protein-bound enrichment (MPE) of L-[ring-^13^C_6_]-phenylalanine was 0.031 ± 0.005% (T-45 min), 0.063 ± 0.004% (T240 resting leg) and 0.073 ± 0.012% (T240 min exercising leg) in the EXP group. In the Control group, MPE was 0.038 ± 0.013% (T-45 min), 0.065 ± 0.013% (T240 resting leg) and 0.069 ± 0.013% (T240 min exercising leg). Muscle free intracellular enrichment (MPE) of L-[ring-^13^C_6_]-phenylalanine was 7.483 ± 0.514% (T-45 min), 9.275 ± 0.642% (T240 resting leg) and 8.636 ± 0.990% (T240 min exercising leg) in the EXP group. In the Control group, MPE was 8.119 ± 0.683% (T-45 min), 8.995 ± 0.411% (T240 resting leg) and 9.579 ± 0.505% (T240 min exercising leg).

### Mixed muscle protein synthesis rate (FSR)

The calculated one-biopsy baseline muscle protein synthesis rate (FSR) was 0.053 ± 0.010 %.h^-1^ and 0.052 ± 0.017 %.h^-1^ for EXP and Control group, respectively.

**Figure 6:**
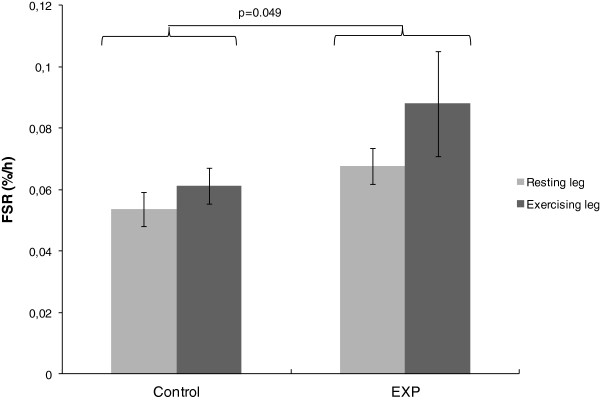
**Mixed muscle protein synthesis.** Mixed muscle protein FSR (means ± SEM) with plasma L-[ring-^13^C_6_]-phenylalanine as precursor, over 4 h after ingestion of the study products EXP (*n* = 9) and Control (*n* = 10). Data are presented for resting leg and exercising leg separately.

Postprandial FSR overall was significantly higher after ingestion of the EXP product (0.078 ± 0.007 %.h^-1^ (EMM ± SE)) than after ingestion of the Control product (0.057 ± 0.007 %.h^-1^ (EMM ± SE)) (*P* = 0.049) (Figure 6). No interaction between treatment and exercise was observed (*P* = 0.52), indicating a similar effect of the EXP vs Control product during rest (*P* = 0.11) and exercise (*P* = 0.14). When comparing the resting and exercising leg, no significant differences are observed between the legs in the Control group (P = 0.0564) and EXP group (P = 0.2908). The estimated mean FSR increase from the calculated baseline in the resting leg was 28% for EXP and 3% for Control. Postprandial FSR values calculated using the muscle free amino acid as precursor indicate a similar pattern as observed with the plasma precursor pool: 0.087 ± 0.006%/h (resting leg) and 0.138 ± 0.045%/h (exercising leg) in the EXP group, and 0.0754 ± 0.025%/h (resting leg) and 0.083 ± 0.008%/h (exercising leg) in the Control group.

When including possible confounding parameters one-by-one in the statistical model, the *P* value for difference in postprandial FSR overall increased from 0.049 (basic model) to 0.15 when including physical activity, and to 0.15 when including dietary protein intake (as en%). Inclusion of LLM in the basic model lowered the *P* value from 0.049 to 0.024, but no significant correlation was observed between postprandial FSR overall and LLM (*P* = 0.82).

**Figure 7:**
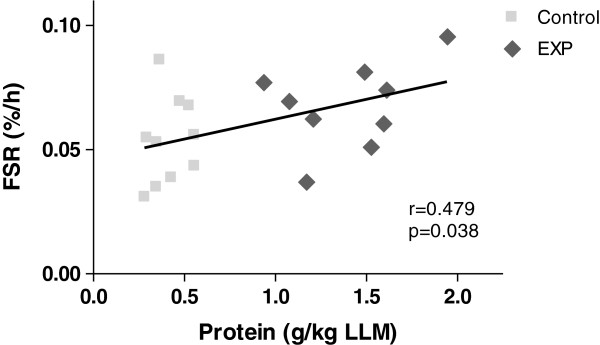
**Correlation between FSR and leg lean mass-corrected protein intake.** Correlation between intake of protein from EXP (21 g) and Control (6 g) per kg leg lean mass (LLM) and postprandial FSR in the resting leg. Pearson’s correlation statistics is shown.

When expressing the intake of total protein from EXP (21g) and Control (6g) per kg LLM, a significant correlation was observed with the resting postprandial FSR (r = 0.48, *P* = 0.038) but not with the exercising postprandial FSR (*P* = 0.29)(Figure 7).

### Possible mediating factors to the FSR effect

Several parameters were included one-by-one in the basic mixed model to identify the factors related to product intake that potentially mediate or attribute to the FSR effect. iAUC leucine and iAUC EAA appeared the strongest mediators, changing the *P* value in the model from 0.049 to 0.99 and 0.85, respectively. Including iAUC AA, maximum leucine and maximum EAA in the statistical model, moderately changed the *P* value to 0.55, 0.52 and 0.49, respectively. NEAA and glucose only slightly mediated the FSR response (*P* value changed to 0.18 and 0.14, respectively), while insulin did not mediate the FSR response (no change in *P* value).

This is also shown by the correlations between postprandial FSR overall and iAUC leucine (r = 0.35, *P* = 0.031), iAUC EAA (r = 0.34, *P* = 0.034), maximum leucine (r = 0.32, *P* = 0.05), and maximum EAA (r = 0.31, *P* = 0.06). iAUC NEAA was not significantly correlated with postprandial FSR overall (*P* = 0.18).

## Discussion

A significantly higher overall postprandial muscle protein synthesis rate was observed after intake of the specifically designed high whey protein, leucine-enriched nutritional supplement, as compared with the isocaloric control product containing milk protein in an amount that is typical for a single serving of a conventional dairy product. In this study using the unilateral resistance exercise model, we did not observe a further enhanced postprandial muscle protein synthesis response.

### Postprandial FSR response and product-related factors

In line with our hypothesis, a nutritional supplement containing 20 g whey protein with added free leucine to a total amount of 3 g leucine resulted in a greater overall mixed muscle protein synthesis rate during the 4-h postprandial period (0.0780 ± 0.0070%/h) than 6g milk protein in an iso-caloric product (0.0574 ± 0.0066%/h). Previous studies showed greater stimulation of postprandial muscle protein synthesis with a higher amount of total leucine (~2.8-3.5 g leucine in a mixture of 6.7 g EAA or added to 20g casein protein) [12,26], a higher dose of EAA (~10-15 g) [7,11,27], or a higher amount of whey protein (~20-35 g) [14,15,28], when compared to a low amount of EAA (7 g), a lower amount of whey protein (10-20 g) or 20 g casein protein alone. Our data extend on this previous work by showing that a combination, i.e. 20 g whey protein, 11 g total EAA and 3 g total leucine, is effective for stimulation of overall muscle protein synthesis in the context of a nutritional supplement that contained also fat and carbohydrates besides protein. In line with previous findings we also observed that an increase in plasma EAA and leucine likely dictates the extent of stimulation of muscle protein synthesis [9,10,12,14,29] while plasma NEAA attribute much less to postprandial muscle protein synthesis [9,10]. The larger amount of carbohydrates in the iso-caloric Control product resulted in a rapid increase of plasma glucose to a higher peak concentration and iAUC compared with the EXP product that contained fewer carbohydrates. However, the insulin response to both products was similar, which is likely due to leucine-stimulated insulin secretion in the EXP product [30,31], and stimulation of insulin release by the high carbohydrate content in the Control product. Adding insulin as a possible mediator in the statistical model indicated that the FSR response was not related to alterations in serum insulin in our study, which confirms that the observed difference in muscle protein synthesis response between the products is at least not due to a difference in endogenous insulin release. The role of insulin on muscle protein synthesis is somewhat debated and inhibition of muscle protein breakdown by insulin may be even more important [32]. In existing studies in healthy older participants, the addition of carbohydrates has not been shown to augment muscle protein FSR [33], and one study demonstrated a blunted FSR response with carbohydrates added to a mixture of amino acids [4]. It can also be hypothesized that a decrease of protein breakdown lowered amino acid availability if no nutrients were given and FSR would then have likely decreased, even though net balance might have improved. An important limitation of the present study design therefore is the absence of protein breakdown measures. In particular in the context of a mixed meal, the total anabolic response to protein is ultimately a function of muscle protein synthesis and protein breakdown as also reflected by a linear relation between protein intake and net protein synthesis [27].

### Postprandial FSR response at rest

The postprandial FSR response was significantly higher after intake of the high whey protein, leucine-enriched product in the overall comparison, but the difference was not significant for the resting condition as such. Lack of statistical power and inter-individual variation in FSR are, at least partly, accountable. Increasing the sample size to 25 participants per group would have increased the power from 40% to 80% to show a postprandial FSR difference between the two groups in rest. A cross-over design or comparison between the groups of the FSR increase (i.e. change from baseline) could have reduced the variation, favoring statistical significant difference between the two groups.

The estimated 3% increase of postprandial FSR with the Control product suggests that a conventional dairy product is not adequate to acutely stimulate muscle protein synthesis in older people, as expected from the protein/EAA content of the dairy product that is below 7 g EAA [11]. The 28% estimated increase of postprandial mixed muscle FSR with the high whey protein, leucine-enriched nutritional supplement seems somewhat less than previously observed values with 2.8 g leucine in a mix of 6.7 g EAA [12], 15 g EAA [7,10], or compared with the difference in postprandial FSR between 20 g whey and 20 g casein protein [14]. However, the effect is in line with the 16% increase from basal with 20 g whey protein in a recent study in older adults [34]. While our study products contained also carbohydrates and fat to mimic conventional nutrition, all previous studies referred to above used supplementation of protein only without the addition of carbohydrates or fat. The combined effect of additional macronutrients on postprandial FSR response to protein intake therefore needs further investigation to define the effect of added carbohydrates and fat.

### Postprandial FSR response with combined exercise

Combined exercise showed a similar effect in both groups (no interaction) in our study, but the variation in FSR response to the combined exercise-nutrition intervention was large. It has previously been observed that the muscle protein synthesis response to exercise alone is impaired in older people [5,6]. But when combined with ingestion of 20 g casein [35] or 90 g beef protein [36], resistance exercise increased mixed muscle protein FSR to a higher level than dietary intervention alone, similar to the young. A recent dose–response study with whey protein (0, 10, 20 and 40 g) in older men showed that at least 20 g of whey was required to increase myofibrillar FSR above exercise alone [28]. Moreover, resistance exercise-stimulated myofibrillar FSR was greater after ingestion of 20 g whey protein than after 20 g casein protein [15]. It is possible that a higher protein amount in the supplement or measuring the synthesis of myofibrillar proteins, which are affected to a greater degree by resistance exercise than other muscle proteins [37], would have resulted in a significant effect of combined exercise. Another aspect demonstrated by Drummond et al. [20] is that, in contrast to young, resistance exercise before ingestion of 20 g EAA only stimulated muscle protein synthesis at 3–6 h post-exercise and not during the first 3 h post-exercise in older people. It is therefore possible that the 4-h interval for studying muscle protein synthesis in our study was too short to show an effect of combined exercise.

### Subject characteristics that potentially influence the FSR response

Interestingly, the inclusion of LLM in the mixed model increased the statistical power in our study. When the protein intake from the supplements was then expressed per kg LLM, a significant correlation was observed with resting postprandial FSR. It is therefore likely that the degree of muscle anabolic response to product supplementation is also dependent of the LLM of the subject. The data seem indicative of a dose–response relation between protein and FSR when LLM is taken into account. However, various dosages of the same protein source and different protein sources should be tested to discriminate between protein amount and source in such relationship. Moreover, we observed that both habitual protein intake (as en%) and physical activity level reduced the statistical power when included in the mixed model. Although both variables were not strong confounders (i.e. *P* value below 0.2 after inclusion in the mixed model), it may point toward potential relevance of these factors in the muscle anabolic response. Physical activity level and composition of the habitual diet have both been suggested to be related to a blunted muscle protein synthesis response to nutritional stimuli [38]. Therefore, the relevance of LLM, habitual dietary protein intake (as en%) and physical activity level for the acute muscle anabolic response in older people needs further study. Especially in sarcopenic older adults, since this group is generally characterized by lower LLM, lower dietary protein intake and a lower physical activity level.

## Conclusions

A specifically designed nutritional supplement high in whey protein and enriched in leucine is more effective than a conventional dairy product to stimulate postprandial muscle protein synthesis overall in healthy older subjects. This effect is attributable to the higher plasma levels of leucine and EAA after intake of the high whey protein, leucine-enriched supplement, while changes in plasma NEAA are of less relevance and plasma insulin increase does not dictate the FSR response. Moreover, the effects on protein breakdown and the impact of other macronutrients on the anabolic effect of protein needs further study, as well as the effect of subject characteristics leg lean mass, habitual protein intake and physical activity level. Finally, this acute effect on postprandial muscle protein synthesis is promising for potential long-term effects on parameters of muscle mass, strength and function in sarcopenic older people. This requires further study.

## Competing interests

This study was financially supported and study products were provided by Nutricia Advanced Medical Nutrition, Danone Research, the Netherlands.

All authors have made substantial contributions to the conception and design of the study, acquisition of data, analysis and interpretation of data, drafting and critically revising the article for intellectual content. Each author has seen and approved the contents of the submitted manuscript.

YCL, RM and SV are employed by Nutricia Advanced Medical Nutrition. YCL has also an affiliation at CTRAL. RRW was a member of the Danone Research Advisory Board at the time of study and received compensation. NEPD has no conflict of interest to declare.

## Authors’ contributions

YCL was involved in writing of the protocol, the statistical analysis plan, data interpretation and drafting the manuscript. NEPD was involved in data interpretation. RM was involved in study coordination, writing the protocol and the statistical analysis plan. SV initiated the study and was involved in data interpretation. RRW was principle investigator of the study, and involved in all aspects of the study. All authors approved the final draft of the manuscript.
